# Supplementary material for: Proteomics of immune cells from liver tumors reveals immunotherapy targets
Source: Cell Genom. 2023 May 30;3(6):100331. doi: 10.1016/j.xgen.2023.100331 (PMC10300607; doi:10.1016/j.xgen.2023.100331)
Supplement: Document S1. Figures S1–S6 [file mmc1.pdf]

Cell Genomics, Volume *3*

Supplemental information

Proteomics of immune cells

from liver tumors reveals immunotherapy targets

Fernando P. Canale, Julia Neumann, Janusz von Renesse, Elisabetta Loggi, Matteo Pecoraro, Ian Vogel, Giada Zoppi, Gaia Antonini, Tobias Wolf, Wenjie Jin, Xiaoqin Zheng, Giuliano La Barba, Emrullah Birgin, Marianne Forkel, Tobias Nilsson, Romina Marone, Henrik Mueller, Nadege Pelletier, Lukas T. Jeker, Gianluca Civenni, Christoph Schlapbach, Carlo V. Catapano, Lena Seifert, Adrian M. Seifert, Silke Gillessen, Sara De Dosso, Alessandra Cristaudi, Nuh N. Rahbari, Giorgio Ercolani, and Roger Geiger

Supplemental Figures

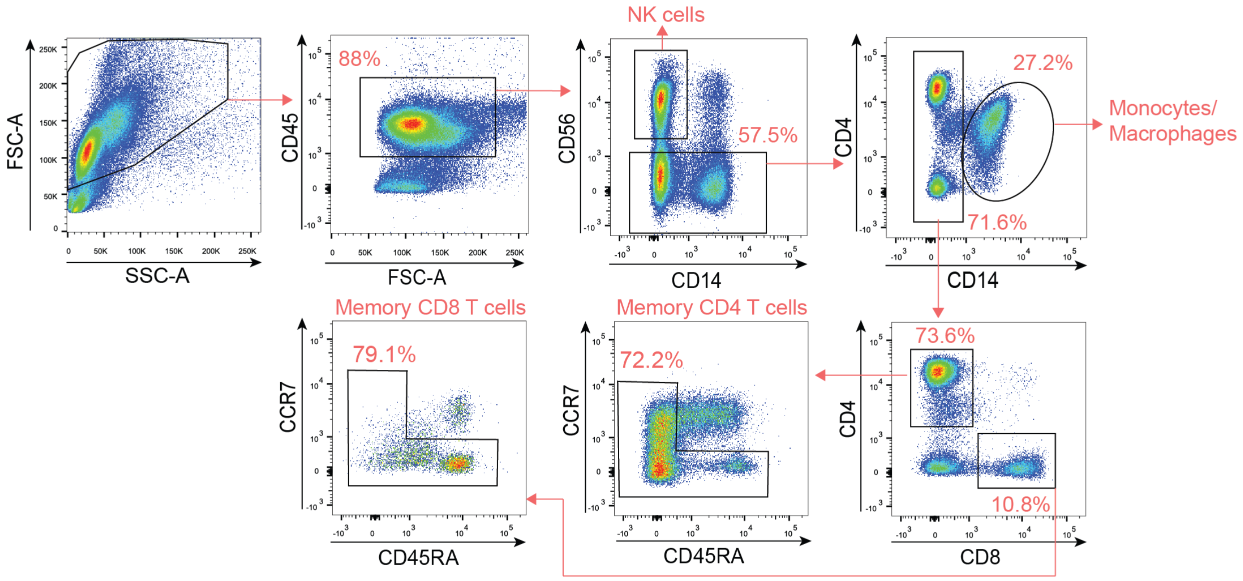

Figure S1: Sorting strategy of immune cells. Related to Figure 1A. Shown are flow cytometry plots and gating strategy to sort CD14$^+$ monocytes/macrophages, total memory CD4$^+$ and CD8$^+$ T cells and CD56$^+$ NK cells. The L-shaped gate on T cells includes CCR7$^+$CD45RA$^-$ central memory T cells, CCR7$^-$CD45RA$^-$ effector memory T cells and CCR7$^-$CD45RA$^-$ EMRA T cells [1].

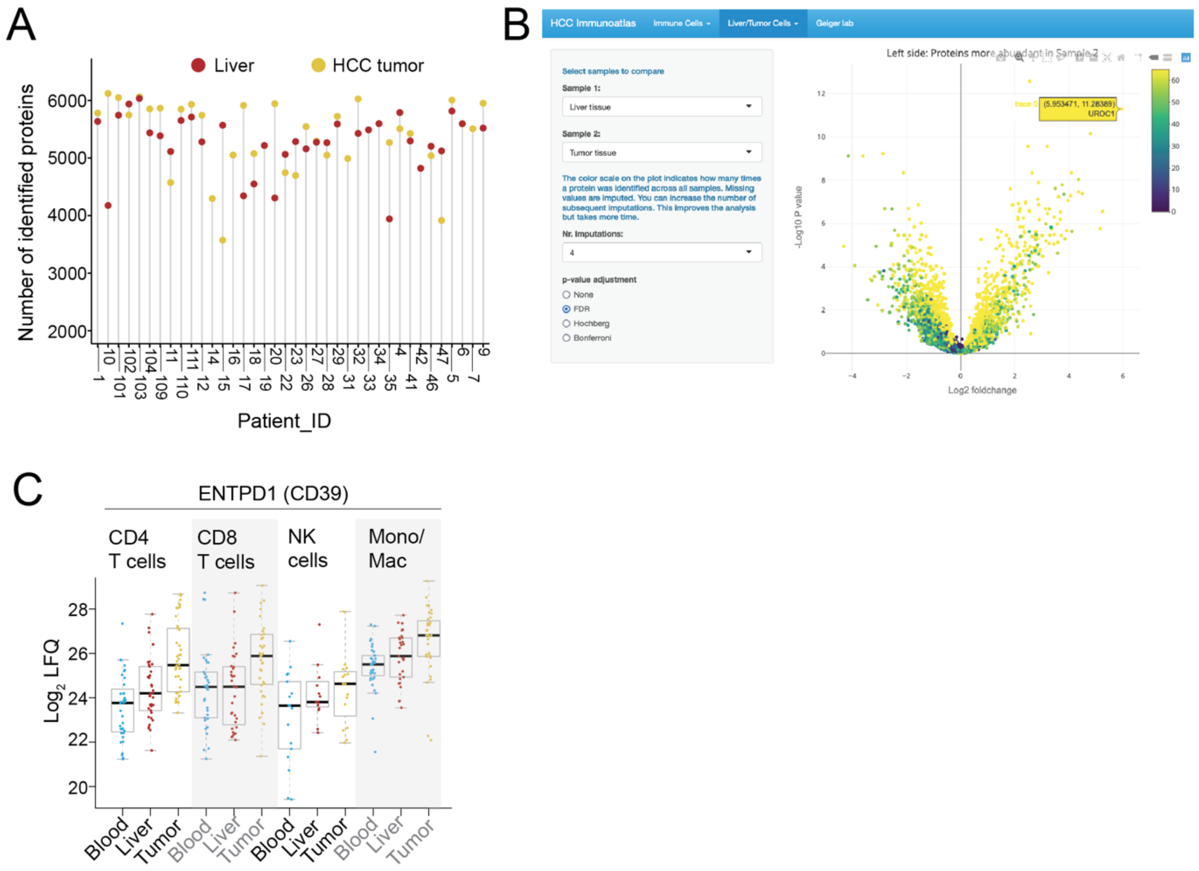

Figure S2: Quality control of liver and tumor proteome data. Related to Figure 2. (A) Tumorous or non-tumorous liver tissue was homogenized and analyzed by LC-MS. Plot shows the number of identified proteins. **(B)** Example from www.immunomics.ch/hcc. Sample groups can be selected for differential abundance analysis, i.e., "Liver tissue" vs. "Tumor tissue". Missing values are imputed and a t-test with Welch correction is performed. The number of cycles of imputation can be adjusted and average p-values and log2 foldchanges are calculated. P-values can be adjusted for multiple comparisons using several methods. The color code shows how many times a protein was identified across all samples. **(C)** Box plots showing the abundance of CD39 protein in different cell types isolated from blood, liver, and tumor tissue. Each dot represents a different patient.

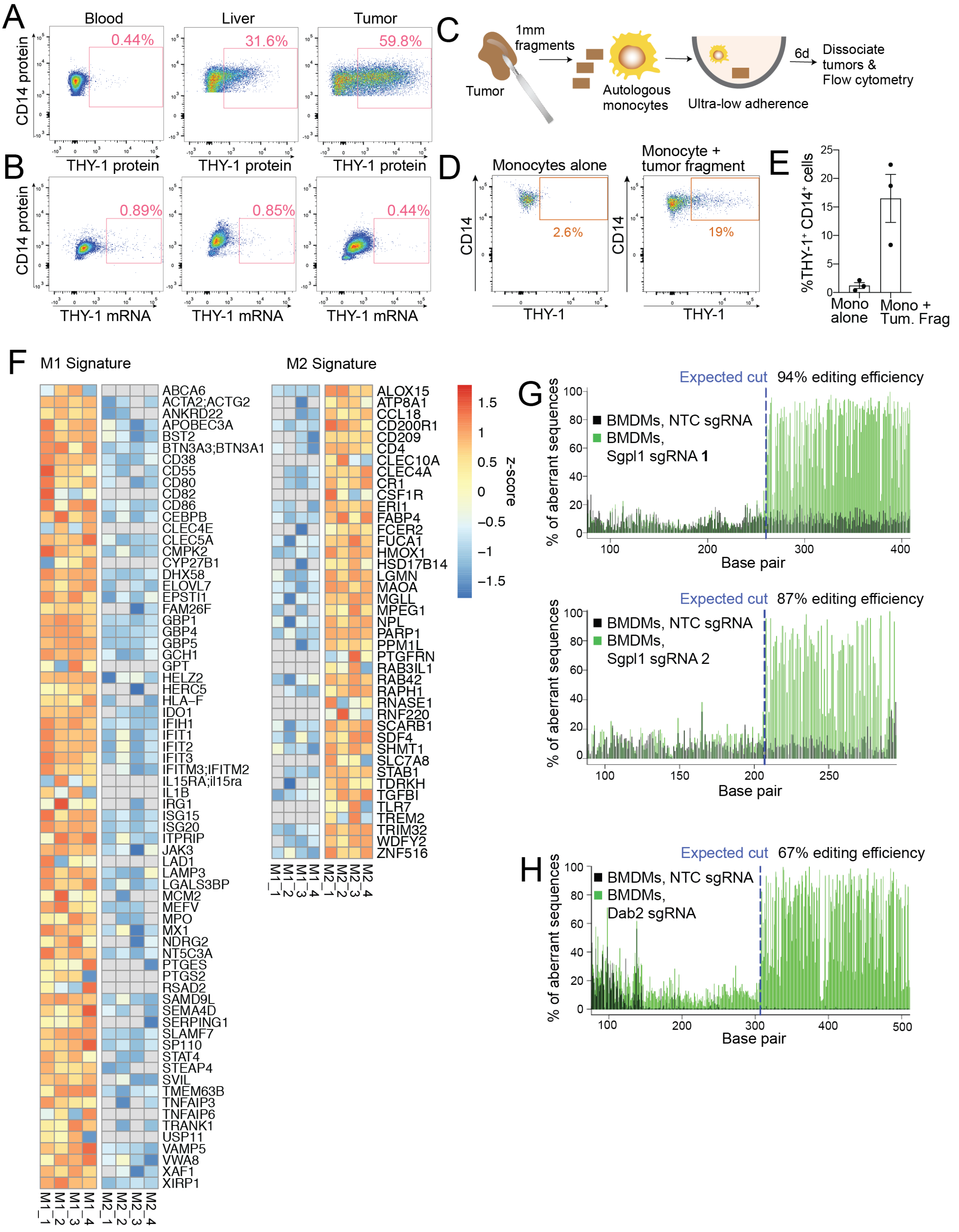

Figure S3: Analysis of macrophages. Related to Figure 3. (A) $CD14^+$ monocytes/macrophages were stained with a monoclonal antibody to THY-1 and analyzed by flow cytometry. Representative scatter plot. **(B)** Same as in (A) but monocytes/macrophages were stained with a PrimeFlow probe targeting THY-1 mRNA. Representative scatter plot. **(C)** Schematic illustration of the co-culture experiment with tumor fragments and autologous monocytes. **(D)** Scatter plot shows percentage of monocytes that acquired THY-1 upon co-

culture with tumor fragments. **(E)** Same as in (D) but quantifications from three independent experiments are shown. **(F)** Signature of human M1 and M2 macrophages. Shown are proteins that are significantly upregulated (*P* value > 0.01, | log2 FC| > 2) from a differential abundance analysis (two-tailed Welch's t test) between proteomes of M1 and M2 macrophages. n=4 **(G)** TIDE analysis of BMDMs edited with two different sgRNAs targeting *Sgpl1*. **(H)** TIDE analysis of BMDMs edited with a sgRNA targeting *Dab2.*

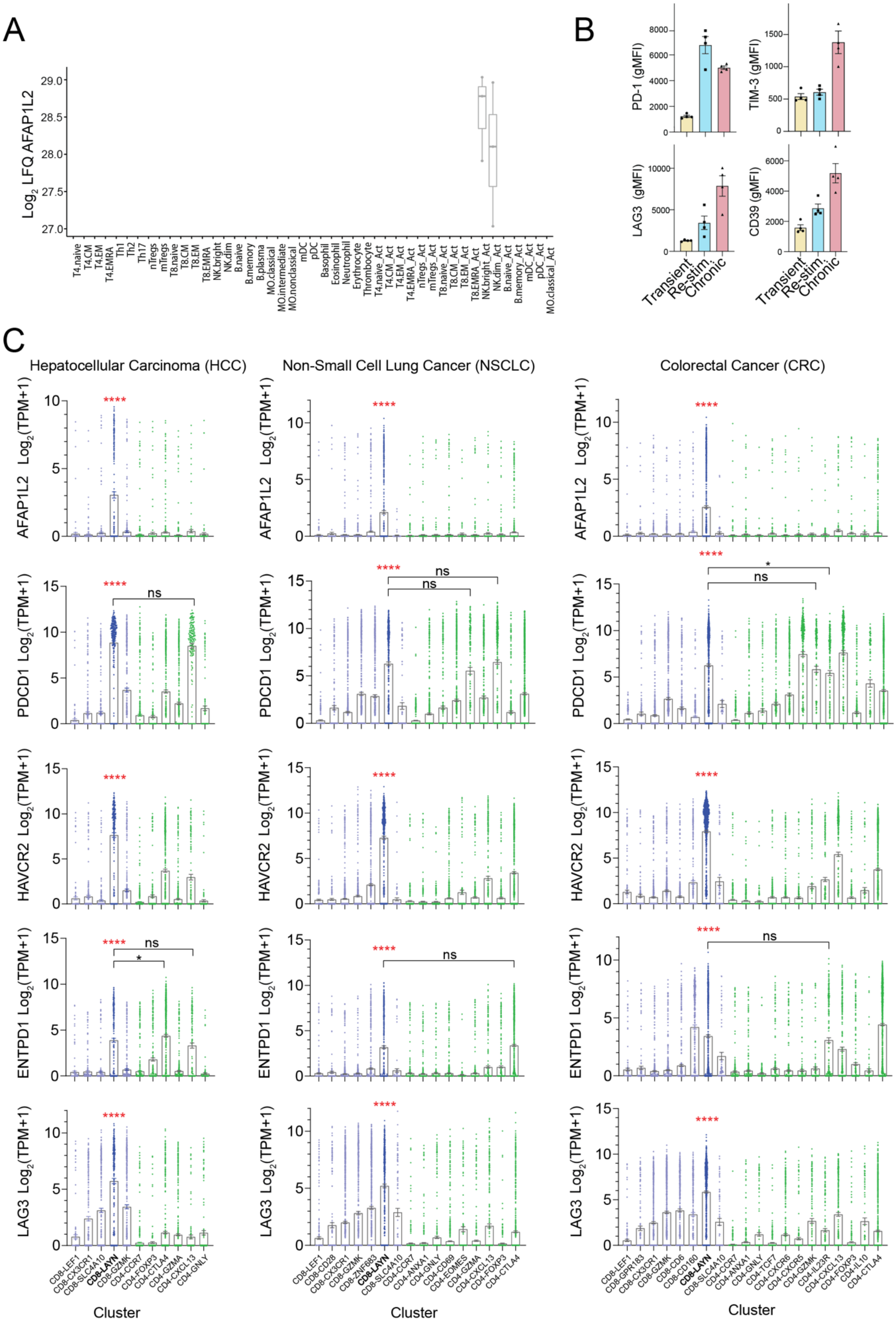

A
B
C
Log2 LFQ AFAP1L2
PD-1 (gMFI)
TIM-3 (gMFI)
LAG3 (gMFI)
CD39 (gMFI)
Transient
Re-stim.
Chronic
Hepatocellular Carcinoma (HCC)
Non-Small Cell Lung Cancer (NSCLC)
Colorectal Cancer (CRC)
AFAP1L2 Log2(TPM+1)
PDCD1 Log2(TPM+1)
HAVCR2 Log2(TPM+1)
ENTPD1 Log2(TPM+1)
LAG3 Log2(TPM+1)
Cluster

Figure S4: AFAP1L2 is expressed in chronically stimulated T cells and NK cells. Related to Figure 4. (A) Analysis of AFAP1L2 protein abundance in different immune cell types. Data are from Rieckmann et al. **(B)** CD8 T cells were transiently stimulated, re-activated, or chronically stimulated as described in Figure 5B. Activation and exhaustion markers were analyzed by flow cytometry. n=4 from four different donors. **(C)** Analysis of AFAP1L2 expression in single cells isolated from tumors of patients with HCC [2], CRC [3] and NSCLC [4]. Clusters are defined in the respective publications. For example, the cluster CD4-CCR7 contains CD4 T cells expressing CCR7, which represent naïve and central memory CD4 T cells. CD8 clusters are shown in blue and CD4 clusters in green. The CD8-LAYN cluster is highlighted. A two-tailed t-test was performed between data in the CD8-LAYN cluster and all other clusters. The red stars (****) indicate a P-value < 0.0001 when comparing the CD8-LAYN cluster to any other cluster. Comparisons for which the P-values were >0.0001 are indicated. n.s. = non-significant; * P < 0.05.

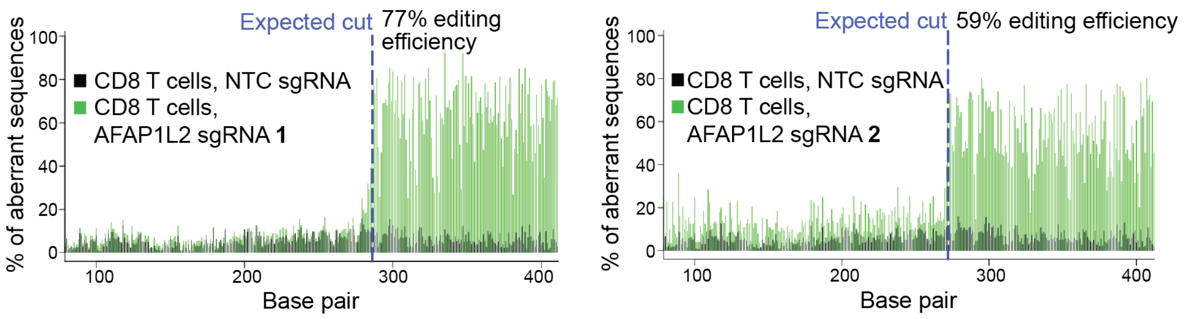

Figure S5: Validation of gene editing in human CD8$^+$ T cells. Related to Figure 5. TIDE analysis of human CD8$^+$ T cells edited with two different sgRNAs targeting *AFAP1L2*.

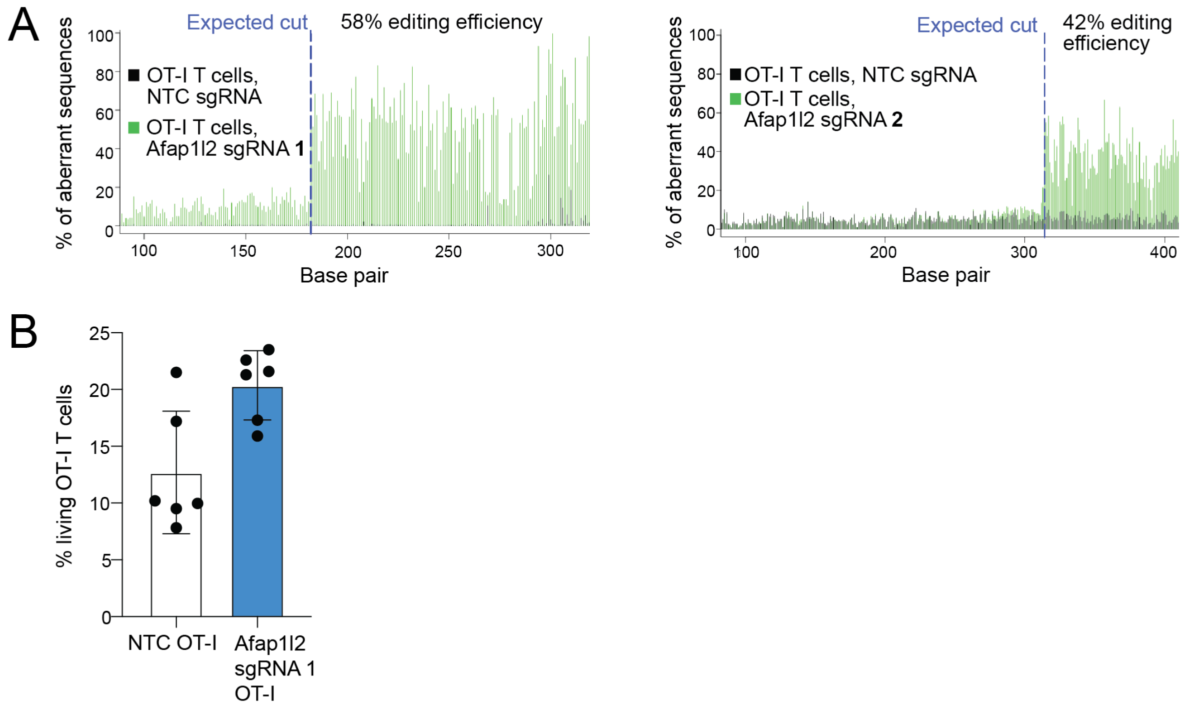

Figure S6: *Afap1l2*-edited CD8^{+} T cells are endowed with a higher survival capacity. Related to Figure 6. (A) TIDE analysis of murine OT-I T cells edited with two different sgRNAs targeting *Afap1L2*. **(B)** NTC or *AFAP1L2*-edited OT-I T cells were rested for 10 days and then activated for 7 days with plate-bound antibodies to CD3 and CD28. The number of live cells was analyzed by flow cytometry. n=6.

Supplemental References

[S1] Sallusto F, Geginat J, Lanzavecchia A. Central memory and effector memory T cell subsets: function, generation, and maintenance. Annu Rev Immunol. 2004;22:745-763. doi:10.1146/annurev.immunol.22.012703.104702

[S2] Zheng C, Zheng L, Yoo J-K, et al. Landscape of Infiltrating T Cells in Liver Cancer Revealed by Single-Cell Sequencing. *Cell*. 2017;169(7):1342-1356.e16. doi:10.1016/j.cell.2017.05.035

[S3] Zhang L, Yu X, Zheng L, et al. Lineage tracking reveals dynamic relationships of T cells in colorectal cancer. *Nature*. 2018;564(7735):268-272. doi:10.1038/s41586-018-0694-x

[S4] Guo X, Zhang Y, Zheng L, et al. Global characterization of T cells in non-small-cell lung cancer by single-cell sequencing. *Nat Med*. 2018;24(7):978-985. doi:10.1038/s41591-018-0045-3
